# The TNFR-RIPK1/RIPK3 signalling pathway mediates the effect of lanthanum on necroptosis of nerve cells

**DOI:** 10.1515/biol-2022-1027

**Published:** 2025-10-14

**Authors:** Bihui Jin, Zhe Ding, Yujiao Sun, Shujuan Gao, Xinyu Sui, Mengping Ding, Xinyi Qu, Linlin Zheng

**Affiliations:** Institute of Molecular Medicine, School of Medicine, Liaodong University, Dandong, 118003, Liaoning, People’s Republic of China; Department of Nursing, School of Medicine, Liaodong University, Dandong, 118003, Liaoning, People’s Republic of China

**Keywords:** La, necroptosis, tumour necrosis factor receptors, mixed lineage kinase domain-like protein, receptor-interacting protein kinases, learning and memory

## Abstract

Lanthanum (La) accumulates resulted in detrimental alterations in the morphology and structure of hippocampal neurons, but the specific mechanism remains unclear. At 49 days after the birth of LaCl_3_-exposed offspring rats, number of Nissl bodies and the neural cell structure in hippocampal tissue was evaluated by Nissl and HE staining; the ultrastructure of hippocampal CA1 zone was observed by electron microscopy. Learning and memory ability of the offspring decreased after LaCl_3_ exposure. Nissl staining showed that in the La-exposed rats, Nissl body number in the hippocampus was significantly decreased, and the cell arrangement was disordered. The ultramicroscopic structure of hippocampal neurons in La-exposed rats showed that the mitochondrial volume was increased; ridges were shorter, decreased in number, and marginally shifted; and the matrix electron density was also decreased. The contents of TNFR1, P-RIPK1, P-RIPK3, and P-MLKL in hippocampal neurons increased significantly as the LaCl_3_ dose increased. La exposure retarded the growth and development of offspring rat, resulted hippocampal nerve cell necroptosis, and impaired spatial learning and memory, which related to abnormal expression of TNFR-RIPK1/RIPK3 pathway.

## Graphical abstract

**Figure:**
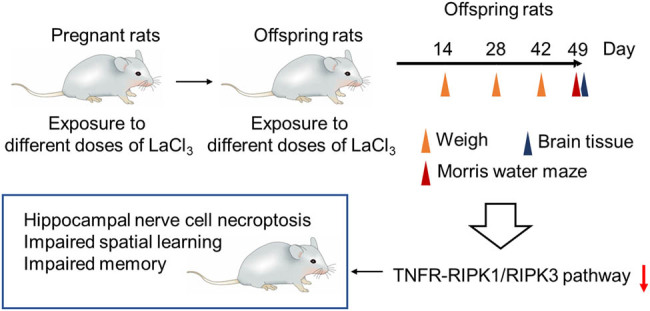

## 1 Introduction

Rare earth elements (REEs) are a collective term for 17 special metal elements that are mostly used in agriculture, industry, and medicine [1]. Numerous studies have confirmed that with the wide application of REEs, REEs in the environment enter the body through various channels, such as the digestive tract, respiratory tract, broken skin, and accumulate in multiple organs [2], and their metabolism is slow. REEs have a certain impact on organs [3]. Research has shown that the central nervous system is the most susceptible system to the toxicity of REEs in humans [4]. REEs can reduce the nerve conduction rate in the central nervous system and affect biological learning and memory [5]. Studies on fetuses have found that REEs accumulate in the uteri of pregnant women, enter the fetus through the placental barrier, and continue to accumulate through breast milk after birth [6]. Exposure to REEs beginning during brain development can continue to impair foetal brain development and cause more serious damage to the nervous system [7]. Lanthanum (La) is a chemically active REE. La is moderately abundant in the Earth’s crust and enters human body through the food chain. Numerous studies have proved that La enters the brain via the blood–brain barrier [8,9]. La accumulates in the central nervous system, resulting in detrimental alterations in the morphology and structure of hippocampal neurons [10], but the specific damaging mechanism is still unclear.

Cell death is usually categorized as programmed death or nonprogrammed death [11]. Recent studies have shown that necroptosis is a typical form of necrosis that occurs in nerve cells [12]. Unlike apoptosis, which is regulated by caspase, this novel cell death mode is regulated by other signals. Research studies have shown that necroptosis can be initiated by the combination of tumour necrosis factor (TNF) and tumour necrosis factor receptors (TNFRs) and is regulated by receptor-interacting protein kinases 1 and 3 (RIPK1 and RIPK3) and mixed lineage kinase domain-like protein (MLKL), which ultimately leads to the occurrence of necroptosis [13,14]. In recent years, studies have shown that hippocampal neuronal cell damage is closely related to this mode of death [15]. Therefore, in this experiment, we evaluated the relationship among necroptosis of hippocampal nerve cells in the offspring rat exposed to LaCl_3_ and the changes in TNFR1, P-RIPK1, P-RIPK3, and P-MLKL protein levels and explored the role of TNFR-RIPK1/RIPK3 necroptosis signalling pathway in La-mediated neurotoxicity.

## 2 Materials and methods

### 2.1 Animals

**Figure 1:**
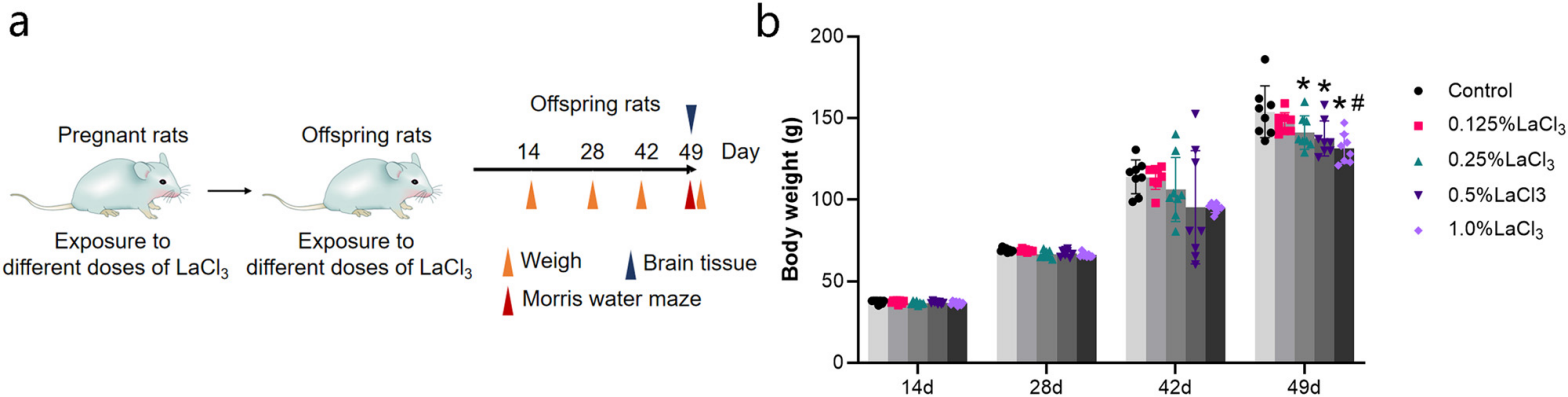
Effects of LaCl_3_ on the weight during growth and development. (a) Schematic illustration for experimental implementation time. (b) Body weight of offspring rats (*n* = 8). *P < 0.05 vs the control group; ^#^ *P* < 0.05 vs the 0.125% LaCl_3_ group.


Forty specific pathogen-free-grade adult Wistar pregnant rats were randomly divided into the control, very low, low, medium, and high-dose groups. According to our previous research [16–19], each group of eight rats was given distilled water or 0.125, 0.25, 0.5, and 1.0% LaCl_3_ solution (LaCl_3_, CAS:20211-76-1 AR, Sinopharm Chemical Reagent Co., Ltd, Shanghai, China) during pregnancy. The offspring ingested LaCl_3_ through the mother’s milk from birth to weaning and continued to ingest LaCl_3_ through drinking water after weaning. All offspring are first born, with half males and half females in each litter. Different rats were used in HE, Nissl, electron microscopy, Morris water maze, and western blot (Figure 1a).

**Ethical approval:** The research related to animal use has been complied with all the relevant national regulations and institutional policies for the care and use of animals, and has been approved by the Local Ethical Committee on Animal Testing at the Liaodong University in China.

### 2.2 Analysis of the growth of offspring rats

The rat offspring were weighed from birth to 49 days and were then killed by anesthesia. The whole brain samples were immediately collected and weighed, then the hippocampal tissues were separated and weighed. The brain tissue ratio and hippocampal tissue ratio were analyzed (brain tissue ratio = brain tissue weight/weight × 100%, hippocampal tissue ratio = hippocampal tissue weight/weight × 100%).

### 2.3 HE staining

Offspring rats (six litters in each group, one in each litter, half male and half female) were euthanized under ether anesthesia. The entire brain was quickly taken, and the brains were fixed with 4% paraformaldehyde. After paraffin embedding, coronal sections corresponding to the cortex, hippocampus, thalamus, and hypothalamus were made, with a thickness of 3 μm. After pasting the slices onto a glass slide and baking, they were routinely dewaxed to water, stained with hematoxylin solution for 3–6 min, and 1% hydrochloric acid alcohol for 1–3 s, followed by a blue promoting solution returning to blue for 5–10 s, and 0.5% eosin for 2–3 min. A Nikon DS-U3 imaging system was used for image acquisition in hippocampal CA1, CA3, and DG zones.

### 2.4 Nissl staining

Tissue sections were routinely deparaffinized to water, and maintained in xylene I for 20 min, xylene II for 20 min, anhydrous ethanol I for 5 min, anhydrous ethanol II for 5 min, followed by 75% alcohol for 5 min. After washing with tap water, Nissl reagent was added to the sections and maintained for 5 min. The tissues were then washed with water, differentiated slightly with 1% glacial acetic acid and washed with tap water to terminate the reaction and control the degree of differentiation under the microscope. After washing, slices were dried in an oven. Tissues were cleared in xylene for 5 min and mounted with neutral gum. A Nikon DS-U3 imaging system was used for image acquisition, and ImageJ software was used to analyze the number of Nissl bodies in nerve cells in hippocampal CA1, CA3, and DG zones. Nerve cell number containing Nissl bodies was measured as Nissl body expression.

### 2.5 Electron microscopy

Approximately 1 mm^3^ of hippocampal tissue from the CA1 zone was collected from 49-day-old offspring, fixed with 3 mL of 2.5% glutaraldehyde for 2 h, and washed with PBS three times for 10 min each. Tissues were fixed with 1% osmic acid for 1 h and washed with PBS three times for 10 min each. Then, the samples were dehydrated with 50% alcohol for 5 min, 70% alcohol for 5 min, 80% alcohol for 5 min, 90% alcohol for 5 min, 90% acetone for 5 min, 100% acetone for 4 min, and 100% acetone for 4 min, for a total of 7 series. The dehydrated samples were maintained in a 1:1 ratio of acetone and epoxy resin 812 at 40°C for 6 h and saturated with pure epoxy resin 812 at 40°C for 4 h. The fixed, dehydrated, and immersed samples were embedded in a special plastic embedding plate, the end to be sliced was aligned with the tip of the plastic embedding plate, and the label was placed at the same time. The embedding agent was added, and the samples were transferred to an oven for polymerization at 40°C for 4 h, 50°C for 2 h, and 90°C for 12 h or more. Then, an ultramicrotome was used to slice 60 nm ultra-thin sections. The cut slices were stained with uranium acetate for 20 min in the dark and then the copper mesh was cut out, washed with double distilled water three times, and blotted dry. Then, the sections were stained with lead citrate for 15 min in the dark. HITACHI HT7700 transmission electron microscopy was used for the observation and comparison of the ultrastructure of hippocampal CA1 region from the offspring in the different groups.

### 2.6 Morris water maze experiments

To assess learning and memory, the training and testing phases of the Morris water maze test were performed in a circular pool (diameter of 160 cm, height of 60 cm, water depth of 30 cm) at 22 ± 2°C. The circular pool was divided into four quadrants. A platform with diameter of 12 cm was placed in the centre of the third quadrant of the pool 2.0 cm below the water surface (40 cm away from the pool wall). The offspring rat was plated in the pool facing the wall at the midpoints of the four quadrants four times for continuous training for 5 days. The time required for each offspring rat reaching the platform was recorded. Offspring rats that reached the platform within 60 s were allowed to stay for approximately 15 s; if the offspring rats did not reach the platform within 60 s, then they were guided to the platform and allowed to stay there for 15 s. After 5 days of training, offspring rats were placed in the maze again, and two tests were performed at an interval of 1 week. One of the tests was the place navigation test. The hidden platform was placed in the centre of the third quadrant. The offspring rats were placed in water at the same position, and the escape latency and total swimming distance (swimming path length) were evaluated. The other test was the spatial probe test. Four hours later, the platform was removed. The offspring rats from each group were allowed to swim in the pool for 60 s, and the time they entered the target quadrant, the amount of time used for the target quadrant, and the path were analyzed.

### 2.7 TNFR1, P-RIPK1, P-RIPK3, and P-MLKL protein expression determination

After hippocampal tissues were sheared and weighed, radio immunoprecipitation assay lysis buffer was added, and the tissue was fully ground on ice. Tissues were transferred to Eppendorf tubes placed in a cryo-centrifuge at 12,000*g* at 4°C for 5 min and the concentrations of supernatants were detected by the bicinchoninic acid assay method (Beijing Dingguo Changsheng Biotechnology CO., LTD, China). The denatured proteins were subjected to vertical electrophoresis on 10% SDS-PAGE (Beijing Dingguo Changsheng Biotechnology CO., LTD, China) for 2 h and 30 min with 30 μg of protein in each runner. After electrophoresis, the proteins were transferred onto a PVDF membrane (Vazyme Biotech Co., Ltd) by electrotransfer for 90 min, and then the PVDF membrane was blocked with 5% skimmed milk at 4°C overnight. The proteins were then incubated with rabbit anti-rat antibodies TNFR1, RIPK3, P-RIPK3, RIPK1, P-RIPK1, MLKL (#32304, #38654, #12840, #24965, #12953, #38674, Signalway Antibody LLC, Maryland, USA), P-MLKL (AP0949, ABclonal Technology Co., Ltd, China), and GAPDH (#21612, Signalway Antibody LLC, Maryland, USA) overnight at concentrations of 1:1,000, 1:1,000, 1:500, 1:500, 1:200, 1:500, 1:200, and 1:5,000 in a 4°C refrigerator. The proteins were then incubated with diluted horseradish enzyme-labeled goat anti-rabbit IgG secondary antibody (ZB-2301, Beijing Zhong Shan Golden Bridge Biological Technology Co., Ltd, China) for 5 h at room temperature. After incubation, the proteins were washed three times with TPBS on a shaker for 15 min each, and then enhanced ECL chemiluminescence solution was added. The membranes were placed in the dark box of a Minichemi chemotypic luminescence imager (SinSage Technology Co., Ltd, China), and images were taken. GAPDH was used as an internal reference control for each membrane. The levels of TNFR1, P-RIPK1, P-RIPK3, and P-MLKL in the hippocampus of offspring exposed to different concentrations of La were determined using ImageJ software.

### 2.8 Statistical analysis

The data were analyzed using SPSS 21.0. One-way analysis of variance was used for data comparison, and the least significant difference test was used for further pairwise comparisons. All results were shown as mean ± SEM. *P* < 0.05 was considered significant.

## 3 Results

### 3.1 Effect of LaCl_3_ on the growth and development of offspring rats

As shown in Figure 1b, there was no statistical difference in body weight at 14 days. As LaCl_3_ dose increased, the weight of the offspring rat showed a downwards tendency from 28 to 49 days. At 49 days, the weights of the offspring rat in 1.0% LaCl_3_, 0.5% LaCl_3_, and 0.25% LaCl_3_ groups decreased in comparison to those of control group (*P* < 0.05). The difference in weight between the 0.125% LaCl_3_ and the 1.0% LaCl_3_ groups was also statistically significant (*P* < 0.05).

Table 1 shows that the whole brain weight of the offspring also showed a downward trend as the LaCl_3_ concentration increased. The difference in weight between the 0.5% LaCl_3_ and 1.0% LaCl_3_ groups and the control group was statistically significant. The weight of the hippocampal tissues of offspring from each group decreased as the La concentration increased. In addition, the brain and hippocampus coefficients of the offspring of each group increased as the La concentration increased without significant difference (*P* > 0.05).

**Table 1:** Effects of LaCl_3_ on brain weight, hippocampus weight, and the brain and hippocampus ratio of the rats

Groups	Brain weight (g)	Hippocampus weight (g)	Brain ratio (%)	Hippocampus ratio (%)
Control	1.625 ± 0.058	0.104 ± 0.010	1.065 ± 0.100	0.068 ± 0.010
0.125% LaCl_3_	1.567 ± 0.062	0.100 ± 0.010	1.066 ± 0.047	0.069 ± 0.006
0.25% LaCl_3_	1.556 ± 0.078	0.098 ± 0.013	1.108 ± 0.096	0.071 ± 0.006
0.5% LaCl_3_	1.528 ± 0.059*	0.097 ± 0.007	1.115 ± 0.072	0.071 ± 0.009
1.0% LaCl_3_	1.456 ± 0.083*^#&$^	0.095 ± 0.012	1.110 ± 0.056	0.073 ± 0.008
df	39	39	39	39
*F*	6.424	0.832	1.874	0.638
*P*	0	0.514	0.137	0.637

Note: *P < 0.05 vs the control group; ^#^ *P* < 0.05 vs the 0.125% LaCl_3_ group; ^&^ *P* < 0.05 vs the 0.25% LaCl_3_ group; ^$^ *P* < 0.05 vs the 0.5% LaCl_3_ group.

### 3.2 Effects of LaCl_3_ on neuronal cells in CA1, CA3, and DG zones of hippocampus of offspring rats

**Figure 2:**
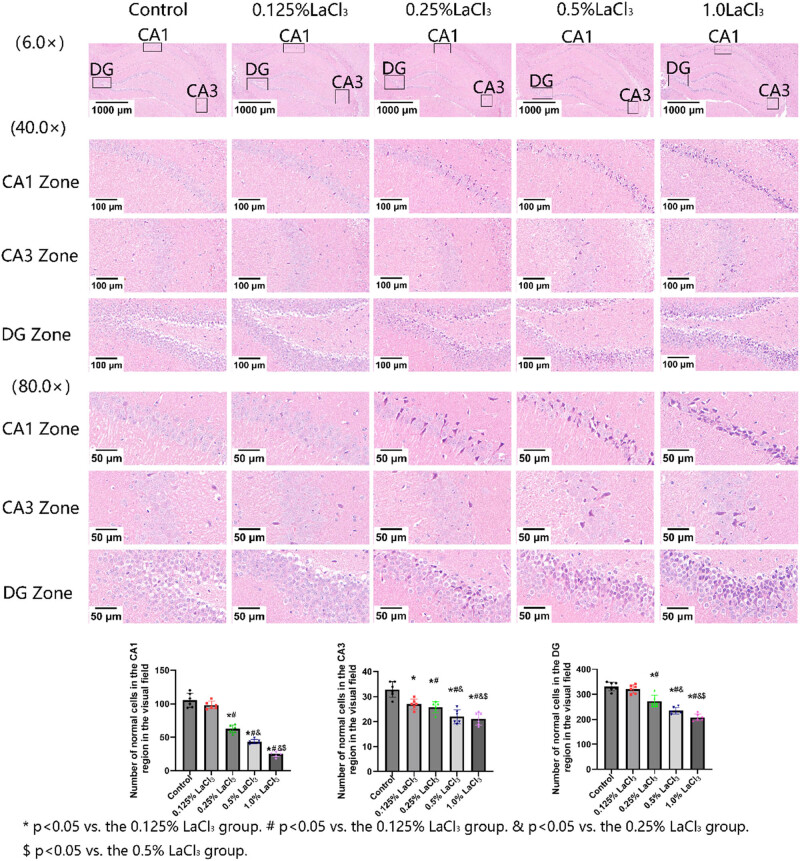
Effects of LaCl_3_ on neuronal cells in the CA1, CA3, and DG zones of the hippocampus (HE, ×400).

As shown in Figure 2, there were circular nucleus with obvious nucleoli, neat cell arrangement, and clear cell boundaries in the nerve cells of control and 0.125% LaCl_3_ group. As the concentration of La exposure increased, the number of nerve cells in the CA1 zone of the 0.25% LaCl_3_, 0.5% LaCl_3_, and 1.0% LaCl_3_ groups gradually decreased. It showed that neuronal cytoplasmic contraction and deformation, nuclear pyknosis, and triangular shrinkage of neuronal cells, resulted in neuronal necrosis in CA1 and DG zones of the 0.25% LaCl_3_, 0.5% LaCl_3_, and 1.0% LaCl_3_ groups. The morphological changes of CA3 cells were not significant.

### 3.3 Effect of LaCl_3_ on Nissl body number in the CA1, CA3, and DG zones of the hippocampus in offspring rats

**Figure 3:**
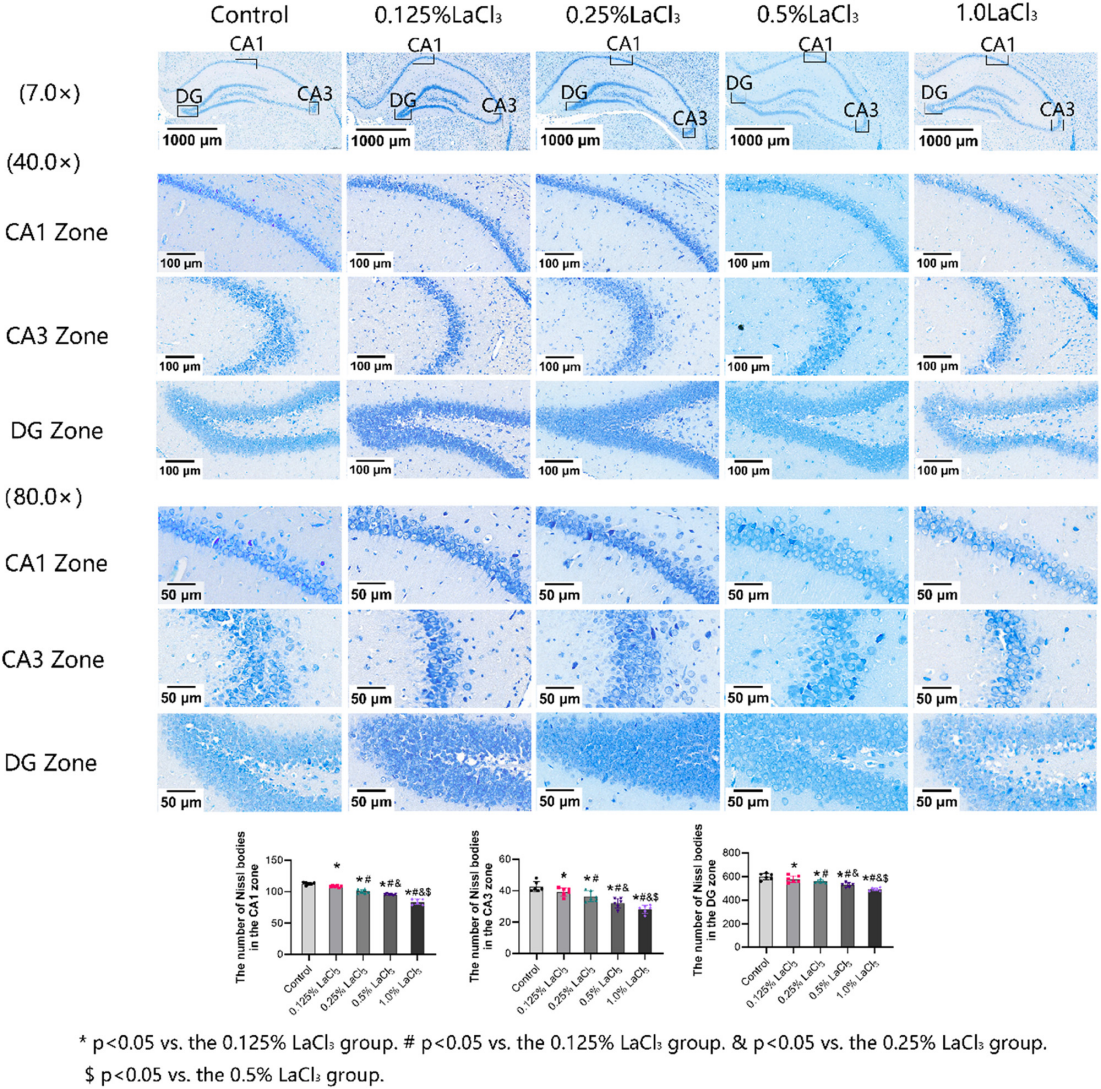
Effects of LaCl_3_ on the number of Nissl bodies in the CA1, CA3, and DG zones of the hippocampus (*n* = 6). *P < 0.05 vs the control group; ^#^ *P* < 0.05 vs the 0.125% LaCl_3_ group; ^&^ *P* < 0.05 vs the 0.25% LaCl_3_ group; ^$^ *P* < 0.05 vs the 0.5% LaCl_3_ group (Nissl dye, ×400).

As revealed in Figure 3, pyramidal cells in the CA1, CA3, and DG zones of the hippocampus were arranged neatly and had a large number of Nissl bodies in control group. As the LaCl_3_ concentration increased, Nissl body number in the hippocampal CA1, CA3, and DG zones of offspring of each La exposure group decreased significantly as the LaCl_3_ dose increased. Nissl body number in hippocampal CA1, CA3, and DG zones was the lowest in the 1.0% LaCl_3_ group, and hippocampal cells were more scattered in the 1.0% LaCl_3_ group in comparison to control group.

### 3.4 Effect of LaCl_3_ on the ultramicrostructure of the hippocampal CA1 area in offspring rats

**Figure 4:**
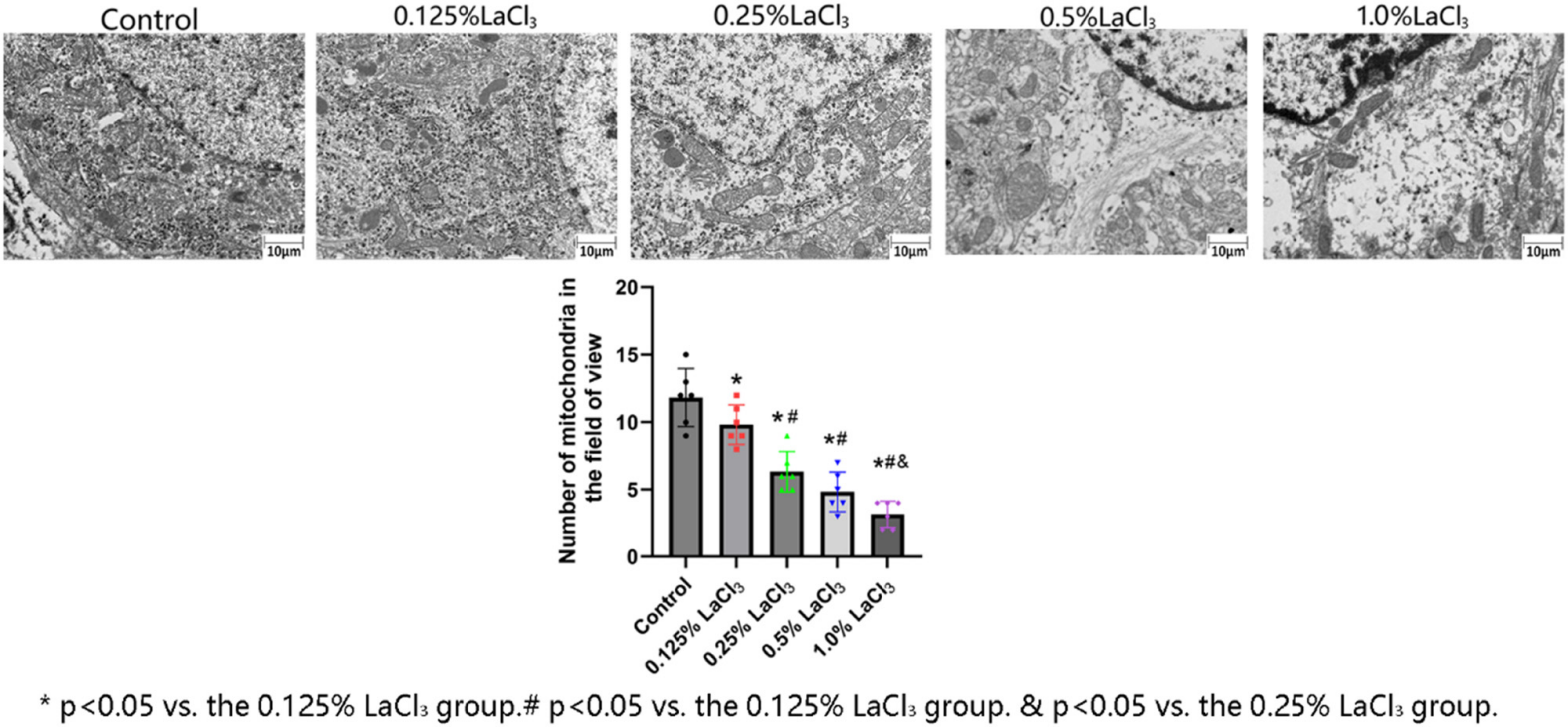
Effects of LaCl_3_ on the ultrastructure of nerve cells in the hippocampal CA1 zone, scale bar = 10 μm.

As shown in Figure 4, the morphology and quantity of organelles in each group were different under the electron microscope. The mitochondria in the hippocampal nerve cells of the control group were normal, as manifested by a large number of mitochondria near the nucleus, normal mitochondrial morphology, a large number of mitochondrial ridges, and the presence of long ridges. As the dose of La increased, the number of mitochondria near the nuclei hippocampal nerve cells decreased, and the morphology of mitochondria gradually changed, as manifested as an increase in mitochondrial volume, a decreased number of shortened ridges and shifting of the ridges to the side, and a decreased matrix electron density. Mitochondria in the 1% LaCl_3_ group even showed swelling and large vacuolation.

### 3.5 Effect of LaCl_3_ on the spatial learning and memory of offspring rats

**Figure 5:**
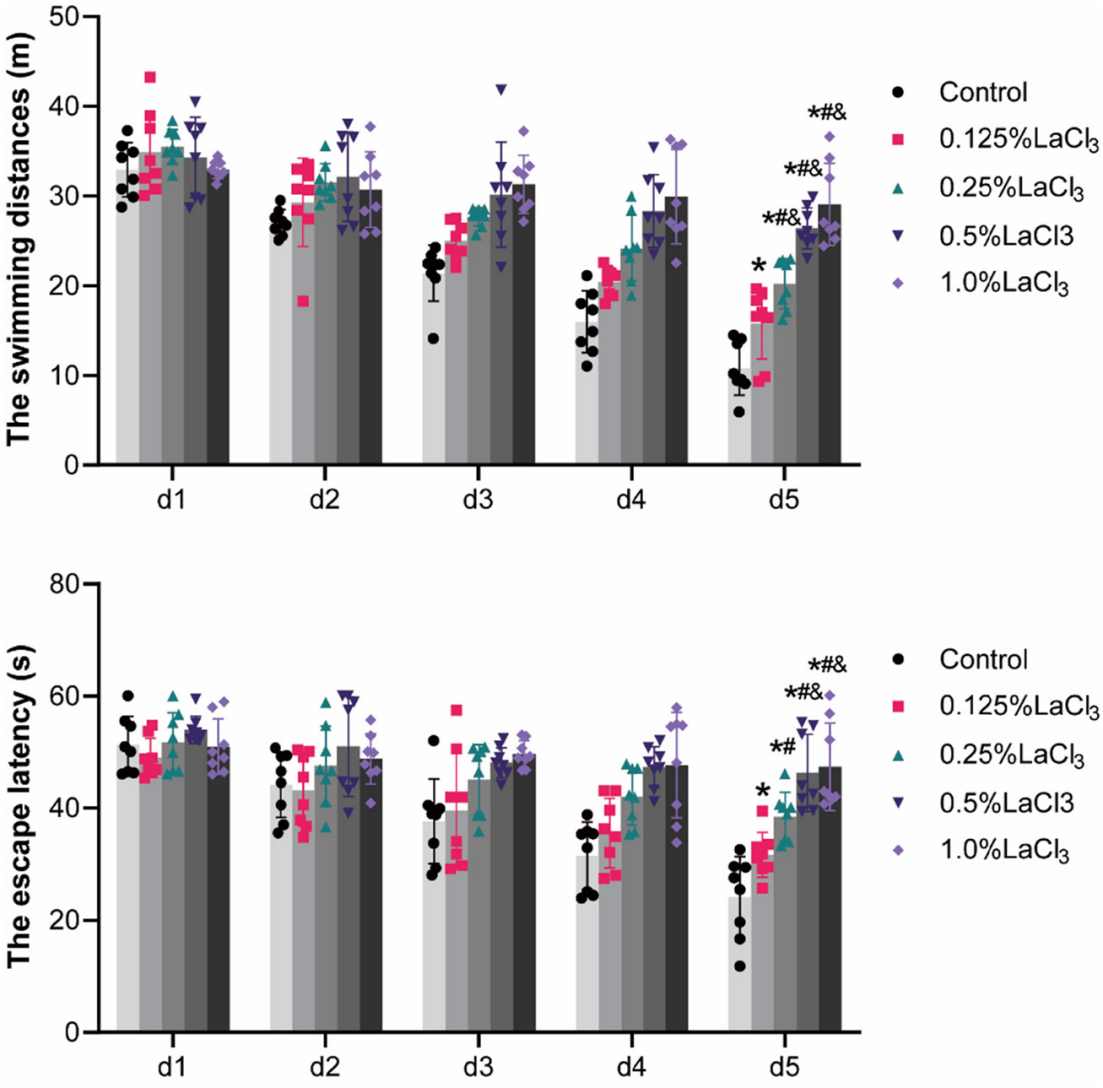
Effect of LaCl_3_ on the swimming distance and escape latency in the Morris water maze during training period. *P < 0.05 vs the control group; ^#^ *P* < 0.05 vs the 0.125% LaCl_3_ group; ^&^ *P* < 0.05 vs the 0.25% LaCl_3_ group; ^$^ *P* < 0.05 vs the 0.5% LaCl_3_ group.

As summarized in Figure 5, during the 5-day training period, the time and swimming distance for the offspring rat to search for hidden platforms gradually shortened. There were differences in escape latency and swimming distance on the last day of the training period, with the lowest in the control group and the highest in the 1.0% LaCl_3_ group (*P* < 0.05).

**Figure 6:**
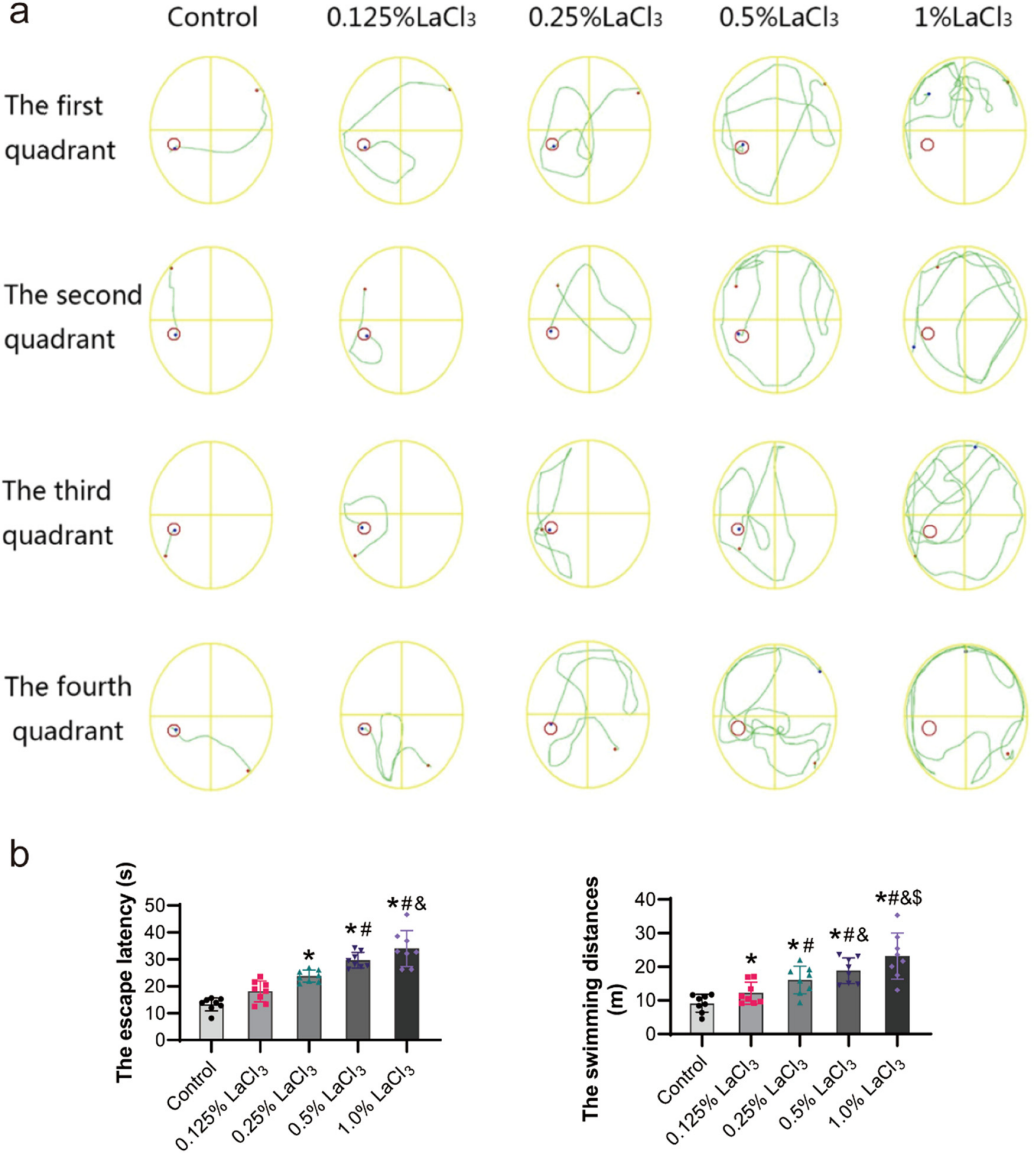
Effect of LaCl_3_ on the performance of the place navigation test in the Morris water maze. (a) Representative search path of the rat offspring in the place navigation test. (b) Representative escape latency and swimming distance in the place navigation test. The escape latency was the average time taken by offspring rats from entering the water to finding the hidden platform (*n* = 8). *P < 0.05 vs the control group; ^#^ *P* < 0.05 vs the 0.125% LaCl_3_ group; ^&^ *P* < 0.05 vs the 0.25% LaCl_3_ group; ^$^ *P* < 0.05 vs the 0.5% LaCl_3_ group.

As summarized in Figure 6, in the place navigation test, the escape latency and swimming distance of the offspring in the 0.25% LaCl_3_, 0.5% LaCl_3_, and 1% LaCl_3_ groups were significantly higher than those of offspring in the control group and were positively associated to the LaCl_3_ dose (*P* < 0.05).

**Figure 7:**
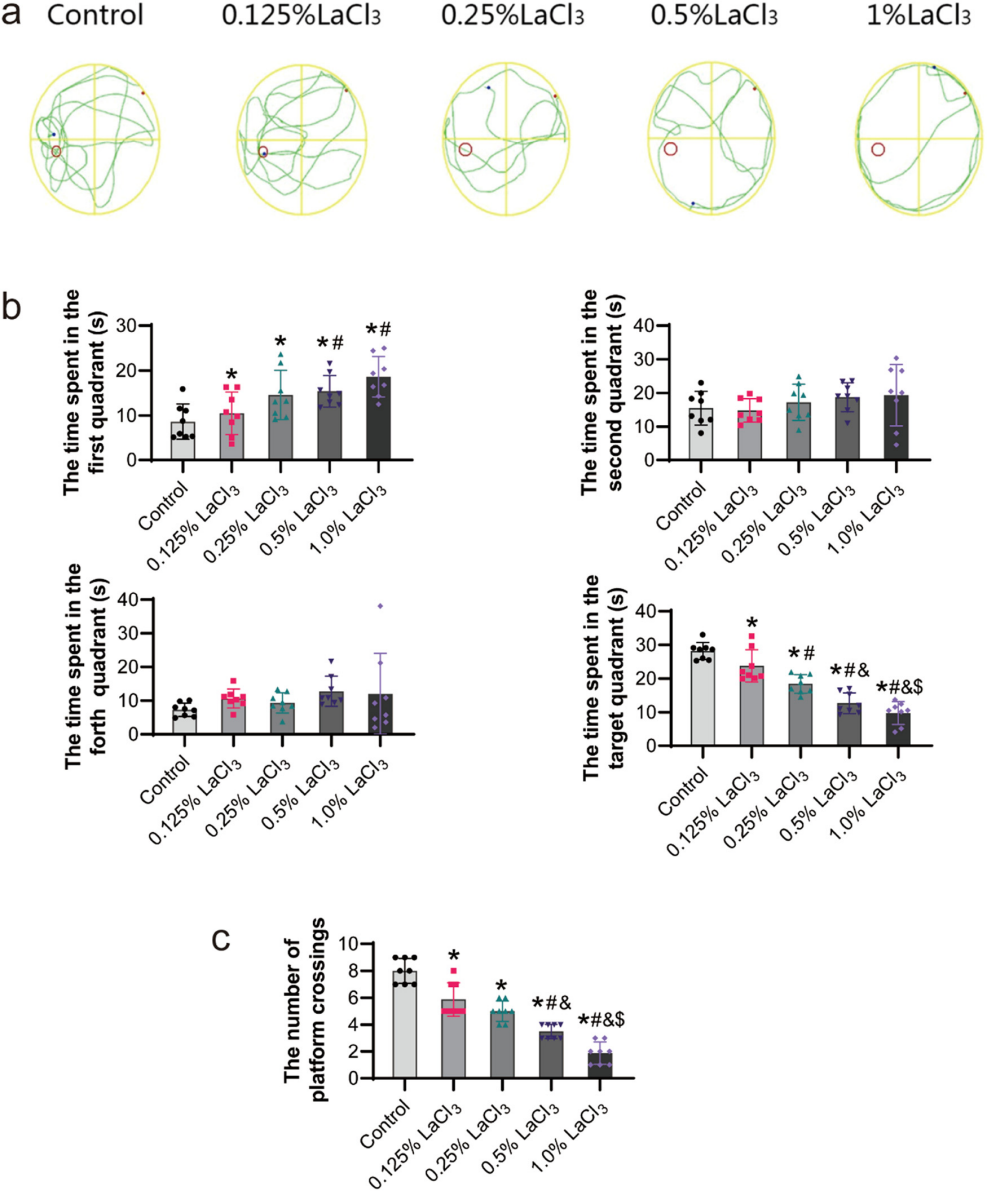
Effect of LaCl_3_ on the performance of the probe test in the Morris water maze. (a) Representative search paths of the rat in spatial probe test. (b) Representative of the time taken in the four quadrants. (c) Representative of the number of platform crossings (*n* = 8). *P < 0.05 vs the control group; ^#^ *P* < 0.05 vs the 0.125% LaCl_3_ group; ^&^ *P* < 0.05 vs the 0.25% LaCl_3_ group; ^$^ *P* < 0.05 vs the 0.5% LaCl_3_ group.

As summarized in Figure 7, in the spatial probe test, observation of the swimming paths of the offspring after the platform was removed revealed that the offspring rats showed different strategies when searching for the original hidden platform: the offspring from the control group showed a strong purpose in looking for the original hidden platform, and they repeatedly traversed the area around the platform, while the tendency of the offspring from the LaCl_3_ exposure groups to look for the original hidden platform showed as the dose of La increased. This also resulted in a difference in the time spent in the first quadrant between the control group and the experimental group. Furthermore, offspring from the 1% LaCl_3_ group seldom entered the platform area. The offspring rats in the 0.25% LaCl_3_, 0.5% LaCl_3_, and 1% LaCl_3_ groups took a markedly shorter time in the target quadrant in comparison to offspring rats of the control group (*P* < 0.05). From the distribution of swimming time of offspring in the four quadrants, the control group spent 56% of the total swimming time in the third quadrant (target quadrant), which was higher than other LaCl_3_ group, with only 18% of the total swimming time in the 1% LaCl_3_ group (*P* < 0.05). LaCl_3_ group spent more time swimming in the first quadrant than the control group, with 35% of the total time in 1% LaCl_3_ group, while 16% in control group (*P* < 0.05). The number of platform crossings in the 0.25% LaCl_3_, 0.5% LaCl_3_, and 1% LaCl_3_ groups declined in comparison to the control group (*P* < 0.05).

### 3.6 Effect of LaCl_3_ on the level of TNF-R1 in the hippocampi of offspring rat

**Figure 8:**
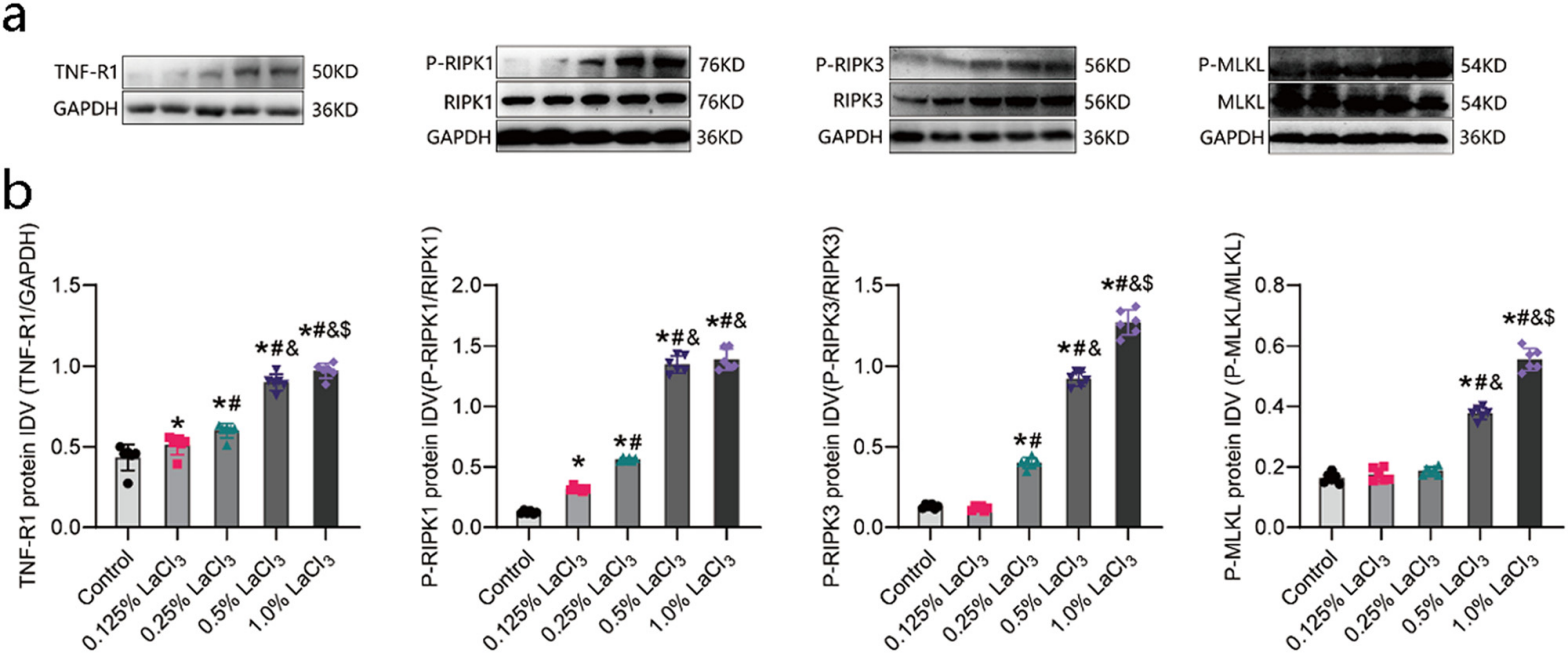
Effect of LaCl_3_ on t levels of TNF-R1, P-RIPK1, P-RIPK3, P-MLKL in the hippocampi of offspring rats (*n* = 6). (a) Representative for the levels of TNF-R1, RIPK1, P-RIPK1, RIPK3, P-RIPK3, MLKL, and P-MLKL proteins. (b) Representative for IDV of TNF-R1, P-RIPK1, P-RIPK3, and P-MLKL. *P < 0.05 vs the control group; ^#^ *P* < 0.05 vs the 0.125% LaCl_3_ group; ^&^ *P* < 0.05 vs the 0.25% LaCl_3_ group; ^$^ *P* < 0.05 vs the 0.5% LaCl_3_ group.

As revealed in Figure 8, LaCl_3_ exposure increased the level of TNF-R1 in the hippocampi of the offspring rat. As the dose of La increased, the level of TNF-R1 in hippocampi of the offspring gradually increased, and TNF-R1 level in each exposure group was different from that of the control group (*P* < 0.05). Moreover, TNF-R1 level in the 1% LaCl_3_ group was higher than that in the 0.5% LaCl_3_, 0.25% LaCl_3_, and 0.125% LaCl_3_ and the control groups, and TNF-R1 level in the 0.5% LaCl_3_ group was higher in comparison to that in the 0.25% LaCl_3_ and 0.125% LaCl_3_ and the control groups (*P* < 0.05).

### 3.7 Effect of LaCl_3_ on the expression of P-RIPK1 in the hippocampi of offspring rat

As shown in Figure 8, LaCl_3_ exposure upregulated the protein level of P-RIPK1 in the hippocampi of the offspring. As the dose of La increased, the protein level of P-RIPK1 in the hippocampi of the offspring gradually increased, and the expression in each exposure group was different from that in the control group. RIPK1 levels were higher in the 1% and 0.5% LaCl_3_ groups compared to the 0.25%, 0.125% LaCl_3_, and control groups. Additionally, P-RIPK1 levels were elevated in the 0.25% LaCl_3_ group relative to the 0.125% LaCl_3_ and control group (*P* < 0.05).

### 3.8 Effect of LaCl_3_ on the expression level of P-RIPK3 in the hippocampi of offspring rat

As revealed in Figure 8, LaCl_3_ exposure upregulated P-RIPK3 level in the hippocampi of offspring rat. As the dose of La increased, P-RIPK3 protein level in the hippocampi of the offspring gradually increased (*P* < 0.05). P-RIPK3 level in the 1% LaCl_3_ group was upregulated than that in the 0.5% LaCl_3_, 0.25% LaCl_3_, and 0.125% LaCl_3_ and control groups. P-RIPK3 level in the 0.5% LaCl_3_ and 0.25% LaCl_3_ group were also upregulated in comparison to the control group (*P* < 0.05).

### 3.9 Effect of LaCl_3_ on the expression level of P-MLKL in the hippocampi of offspring rat

As revealed in Figure 8, LaCl_3_ exposure upregulated the P-MLKL expression in the hippocampi of offspring rat. As the dose of La increased, the P-MLKL level in the hippocampi of the offspring increased gradually (*P* < 0.05). P-MLKL level in the 1% LaCl_3_ group was higher than that in the 0.5% LaCl_3_, 0.25% LaCl_3_, and 0.125% LaCl_3_ groups and the control group, and P-MLKL level in the 0.5% LaCl_3_ group increased than that in the 0.25% LaCl_3_ and 0.125% LaCl_3_ and control groups (*P* < 0.05).

## 4 Discussion

In daily life, La can enter organism via contact or the food chain, especially in areas rich in REEs. A large number of population surveys have shown that it is difficult to excrete La through metabolism after it enters the body. When La accumulates in the nervous system, it exerts a certain toxic effect [20]. Therefore, La is often used to explore the role of REEs on the central nervous system [4,5,20]. According to reports, when La is ingested by growing offspring rats through drinking water, it causes growth retardation [20]. In this experiment, the weight of the offspring rats from the La exposure groups and the weight of the offspring rats from the control group were significantly different, demonstrating that LaCl_3_ affects the normal growth and development of offspring rats.

Hippocampal Nissl bodies are special nerve cell structures that are widely distributed in neurons. Their main function is to synthesize proteins and renew some cell components. The large size and large number of Nissl bodies can reflect the ability of nerve cells to synthesize proteins. In the central nervous system, La can accumulate in the hippocampus by passing through the blood–brain barrier, thereby causing damage to nerve cells [21]. In this study, hippocampal pyramidal cells in the hippocampal CA1, CA3, and DG zones were arranged neatly in the control group, and the cytoplasm contained a large number of Nissl bodies. However, in the offspring rats exposed to La, Nissl body number in the CA1, CA3, and DG regions of the hippocampus declined to varying degrees, the number of hippocampal neurons was reduced, and the cell arrangement was disordered. These performances indicate that the accumulation of LaCl_3_ in offspring has a toxic effect on the hippocampal cells during the growth and development period.

HE staining was used to detect the changes of hippocampal structure. Although the cellular changes in the CA3 zone were not significant, it was found that La exposure resulted in neuronal cytoplasmic contraction and deformation, nuclear pyknosis, and triangular shrinkage of neuronal cells, leading to neuronal necroptosis in CA1 and DG zones of the 0.25% LaCl_3_, 0.5% LaCl_3_, and 1.0% LaCl_3_ groups.

The ultrastructure of hippocampal CA1 area in offspring rat was observed. It was found that as the exposure dose of La increased, the nucleus of nerve cell showed pyknosis, organelle swelling in the cytoplasm, decreased number and abnormal morphology of mitochondria, and even rupture of neuronal cell membrane and overflow of organelles. The results of electron microscopy further demonstrate that La chloride causes substantial damage to hippocampal neurons. The higher the dose of LaCl_3_, the more severe the necroptosis of hippocampal neurons.

The Morris water maze experiment involves directional navigation and spatial probe tests. These two behavioural tests are commonly used to test and evaluate the learning and memory of offspring rats. Here, offspring rats from the control group showed clear goals and found the hidden platform in the two tests, but as the dose of LaCl_3_ gradually increased, the swimming trajectories of the offspring rats became more irregular. The offspring rats in the 1% LaCl_3_ group showed irregular swimming trajectories or swam in circles. Learning in Morris water maze is a stimulating behavior in harsh environment. During the research process, we tried our best to maintain a quiet and dim environment, appropriate water temperature, reasonable training time and frequency for the Morris water maze, etc., in order to minimize adverse external stimuli and reduce the possible anxiety effects of the external environment on the animals. But the Morris water place experiment itself is still a stressful event for animals, especially for the experimental group of rats. Due to the influence of La, learning and memory abilities decline in the experimental groups, and the rats cannot find the underwater platform for a long time, which increases their stress and may cause anxiety, showing a thigmotaxis tendency and swimming against the maze wall. All the above results indicate that La-exposed offspring rat exhibits significantly impaired memory and learning after hippocampal nerve cell necroptosis.

Necroptosis caused by changes in the TNFR-RIPK1/RIPK3 pathway is an important mechanism underlying nervous system damage. Necrosis, as a programmed cell death, is initiated by TNF-α. TNF-α outside the cell binds to TNFR1 on the cell membrane, activates TNFR1, and signals to TNF-related proteins (TNFR-associated death domain [TRADD]) and RIPK1 with the same type of death domain (DD) in the cell through the DD. Furthermore, TRADD also recruits TNFR-associated factor 2, intracellular apoptosis protein inhibitor 1/2 (cIAP1/2), and ubiquitinase to form complex I [22,23]. In complex I, RIPK1 relies on its three domains, namely, the intermediate domain, the amino-terminal kinase domain (KD), and the carboxyterminal DD [24], to regulate cell survival. The occurrence of necroptosis mainly depends on the KD domain of RIPK1 [25]. When RIPK1 dissociates from complex I and the ubiquitination of RIPK1 is blocked, RIPK1 dissociates from complex I and forms complex II together with FADD and Caspase8. If Caspase-8 activity is not suppressed at this time, Caspase-mediated apoptosis occurs [26,27]. In contrast, if Caspase-8 activity is suppressed at this time, the Caspase signalling pathway is inhibited, and RIPK1 in complex II interacts with the RIP homotypic interaction motif domain of RIPK3 through the homotypic domain in the C-terminus to form a RIPK1–RIPK3 complex [28]. The formation and phosphorylation of this complex are key and specific steps in necroptosis [29]. Phosphorylated RIPK3 in the complex recruits MLKL. When MLKL is recruited to necrotic cells, RIPK3 phosphorylates the 357th threonine residue and the 358th serine residue of MLKL. The N-terminal death effect domain 4HBD of MLKL is exposed, and oligomers are formed from monomers [30–32] and transferred from the cytoplasm to the cell membrane, causing the cells to swell and ultimately rupture and die, leading to leakage of cell contents and completing the necroptosis process [21,33].

TNFR1 is the initial receptor protein of the signalling pathway, and TNFR is of great significance to the survival of nerve cells [27]. According to the results, TNFR1 level in the hippocampi of 49-day-old offspring increased as the dose of La increased, indicating that La promoted this pathway to a certain extent.

RIPK1 is a key protein that inhibits learning and memory resulting from necroptosis of hippocampal nerve cells and determines the mode of cell death. Relevant experiments have shown that the RIPK1 inhibitor Nce-1 can alleviate pathological changes in a major cerebral artery occlusion model of stroke [34]. Zhang et al. proved that phosphorylated RIPK1 participates in TNF-α-induced necroptosis [35]. Therefore, the level of P-RIPK1 can also indicate the occurrence of necroptosis. Here, P-RIPK1 level in the hippocampi of offspring rats from the 0.5 and 1% La-exposed groups was markedly higher than that in offspring rats from the control group. It is speculated that there is a basis for necroptosis of hippocampal cells of La-exposed offspring rat.

RIPK3 is a key protein involved in RIPK1-related reactions and in necroptosis. Studies have shown that rats in which RIPK3 is inhibited have the ability to resist necroptosis [36]. The P-RIPK3 complex forms after binding to RIPK1, and phosphorylation of this complex leads to the recruitment and phosphorylation of the MLKL protein, leading to necroptosis of hippocampal nerve cells. In this experiment, as the dose of La increased, the content of P-RIPK3 increased. The P-RIPK3 level in the hippocampus in the 1% LaCl_3_ group was upregulated in comparison to the control group, which indicated that there was a key protein complex for necroptosis formed through the TNFR-RIPK1/RIPK3 signalling pathway in the hippocampi of the offspring.

It is known that in necroptosis, MLKL can change the permeability of the cell membrane leading to cell lysis and death, and the activated phosphorylation state of MLKL was monitored in the programmed necrotic cells undergoing programmed necrosis [37]. The MLKL protein is downstream of RIPK1/RIPK3, and necroptosis is ultimately caused by MLKL-related pathways. P-MLKL is considered a marker of TNF-driven necroptosis [38]. In this experiment, the P-MLKL content in the hippocampus in the La-exposed groups, especially in the 0.5% LaCl_3_ and 1% LaCl_3_ groups, was markedly increased (*P* < 0.05). A higher level of P-MLKL indicates a higher degree of necroptosis, which further suggests that neuronal necroptosis in the hippocampi of the offspring was increased, in turn leading to abnormal learning and memory functions.

Based on previous studies and the results of this experiment, LaCl_3_ has a significant effect on offspring rat from birth to 49 days. The higher the dose of La was, the slower the growth and development of the offspring rat. There were fewer Nissl bodies in the hippocampal CA1, CA3, and DG zones of offspring rats. It was shown that neuronal cytoplasmic contraction and deformation, nuclear pyknosis, and triangular shrinkage of neuronal cells in the hippocampal CA1 and DG zones after La exposure. Furthermore, ultrastructural microscopy revealed structural damage to neural organelles, characterized by cytoplasmic swelling of neuronal organelles, a reduction in mitochondrial count, and morphological abnormalities. These evidence suggest that necroptosis occurred in hippocampal nerve cell. The greater the impairment of learning and memory, the greater the levels of necroptosis-related factors TNFR1, P-RIPK1, P-RIPK3, and P-MLKL in the hippocampus. Based on the above results, it can be speculated that the REE La causes hippocampal nerve cell necroptosis, which is related to the TNFR-RIPK1/RIPK3 necroptosis signalling pathway.
